# RSV Replication, Transmission, and Disease Are Influenced by the RSV G Protein

**DOI:** 10.3390/v14112396

**Published:** 2022-10-29

**Authors:** Harrison C. Bergeron, Ralph A. Tripp

**Affiliations:** Department of Infectious Diseases, College of Veterinary Medicine, University of Georgia, Athens, GA 30605, USA

**Keywords:** RSV, replication, fitness, G protein, immunity, vaccines, transmission

## Abstract

It is important to understand the features affecting virus replication, fitness, and transmissibility as they contribute to the outcome of infection and affect disease intervention approaches. Respiratory syncytial virus (RSV) is a major contributor to respiratory disease, particularly in the infant and elderly populations. Although first described over 60 years ago, there are no approved vaccines and there are limited specific antiviral treatments due in part to our incomplete understanding of the features affecting RSV replication, immunity, and disease. RSV studies have typically focused on using continuous cell lines and conventional RSV strains to establish vaccine development and various antiviral countermeasures. This review outlines how the RSV G protein influences viral features, including replication, transmission, and disease, and how understanding the role of the G protein can improve the understanding of preclinical studies.

## 1. Overview of RSV

Understanding the features that contribute to RSV replication, fitness, and transmissibility is important for understanding the outcomes of infection and disease intervention approaches. RSV infects young children, with many experiencing more than one infection by 2 years of age [1,2]. Globally, RSV causes acute respiratory infection in infants and young children, leading to >60,000 in-hospital deaths and >3 million hospital admissions per year in children <5 years old [3]. The RSV disease burden in high-risk adults with chronic medical conditions is high and similar to non-pandemic influenza [4]. The RSV genome is a negative-sense single-stranded RNA, is 15.2 kb in length, and contains 10 genes encoding 11 proteins [5]. The RSV G and F proteins are the two major surface proteins and have key roles in virus entry, replication, and immune modulation. The attachment G protein binds to ciliated respiratory epithelial cells, and the F protein mediates viral fusion with the target cell membranes [6,7]. The F protein is a major target for antiviral drug and vaccine development, and both the G and F proteins are the antigens targeted by neutralizing antibodies induced by infection. The G protein is produced either as a membrane-bound or secreted protein (e.g., soluble G) that mediates immune evasion [8,9]. The F protein is a trimer in the infected cell membrane in its prefusion form but refolds into a post-fusion form. The SH protein is a minor surface protein in RSV and is expressed at low levels [10]. RSV F, G, and SH proteins can form an oligomeric complex within infected cells, and depending on the host cell, exist as oligomeric forms on the surface of RSV [11,12,13]. RSV is divided into two antigenic groups, i.e., A and B, according to the epitope differences mainly in the G protein [14]. The G protein is the most variable protein [15]. Despite differences in the G protein, it contains a central conserved domain (CCD) and is a CX3C chemokine motif that lacks glycosylation and functions as a chemokine mimic [16,17].

RNA viruses such as RSV replicate using low-fidelity polymerases leading to mutation rates that result in a virus cloud with a distribution of mutants [18]. The spontaneous mutations may alter virus replication leading to the emergence of variant viruses with a selective advantage, i.e., increased fitness [19]. RSV variants can also arise due to selective pressures often by the growth of the virus in the presence of antibodies. These fitness differences in RSV have been linked to nucleotide substitutions in the F gene mediated by anti-F protein antibodies and perhaps mutations occurring elsewhere in the genome [20,21]. Substantial efforts to understand the mechanisms by which virus fitness is increased or decreased are important in vaccine design [14]. Fitness outcomes are measured by both biological (plaque assay) and molecular techniques (nucleotide sequence determination). To understand RSV fitness, one must consider the important steps required in replicating the virus, i.e., binding host cell receptors, entry, virus transcription, translation, and genome replication. The cycle of RSV replication begins with G protein-mediated attachment to the apical surface of polarized, ciliated airway epithelial cells [15], and is facilitated by the G protein CX3C binding to CX3CR1, as well as potentially other cell surface molecules [22,23]. Viral entry is enabled by F protein-mediated membrane fusion [24]. Transcription and replication occur in the host cell cytoplasm in viral inclusion bodies [25]. The viral RNA is transcribed by the viral RNA-dependent RNA polymerase complex and into the positive-sense anti-genome intermediates needed for the replication of new negative-sense genomes for virus packaging [26], where the assembly of RSV occurs at or near the plasma membrane [27].

## 2. RSV Infection Is Cell-Type Dependent

RSV infection occurs following virus inhalation, leading to small foci of infection on the apical surface of respiratory airway cells [28]. This process stimulates the pattern recognition receptors (PRRs), primarily retinoic acid-inducible gene I (RIG-I), but also several Toll-like receptors including TLR2 and TLR4 [29,30,31,32,33]. The RSV F protein has been shown to activate cells through TLR4 [30], whereas the RSV G protein modifies and reduces TLR activation [29,34], and infants with TLR4 SNPs have been shown to be at greater risk for severe RSV disease [35]. TLR and PRR activation initiates host innate immune responses that include expression of type I and III IFNs, IFN-stimulated genes (ISGs), and a variety of proinflammatory cytokines and chemokines [36,37]. To gain a better understanding of RSV and the host response to infection, most studies have typically examined RSV strain A infection of continuous or transformed cell lines such as Hep-2 cells, A549 cells, and Vero cells; however, the results often do not foretell the outcomes from primary human epithelial cell systems or with clinical RSV isolates, particularly as there are several RSV proteins that modify nonspecific (e.g., interferons) and specific (e.g., antibodies) factors affecting virus replication.

Hep-2 (human epithelial) cells were originally derived from a human larynx carcinoma and have shown evidence of contamination with HeLa cells [38]. A549 cells are type II lung carcinoma epithelial cells and were derived from an epithelial carcinoma [39]. One study showed important RSV subgroup-dependent differences in the viral gene expression between RSV-infected Hep-2 and A549 cells [40]. Specifically, Hep-2 cells had increased viral titers and host gene expression compared to RSV-infected A549 cells, suggesting that the origin of the host cell has a dominant effect, whereas the infecting RSV strain has a smaller role in the outcome of an RSV infection. These data revealed important differences in the host response to RSV infection between two widely used continuous cell lines, suggesting the need to evaluate relevant human cells and clinical viruses. Vero cells, derived from the kidney of an African green monkey, are one of the more commonly used continuous cell lines [41]. These cell lines have known differences in RSV propagation outcomes in which the altered expression of viral proteins can occur, as well as differences in glycosylation [12,40]. Most changes have been associated with the cell-specific glycosylation differences of the G protein, which have been demonstrated using lectins and carbohydrate-specific antibodies [11]. For example, when RSV is propagated in Vero cells, the predicted molecular mass of the G protein is ~55 kDa [12], and in Hep-2 cells, the G protein is ~90 kDa, but when RSV is propagated in primary cultures, the G protein is ~170 kDa [42]. In addition, it is important to be cognizant that RSV grown in Vero cells may contain a truncated attachment protein that alters RSV infectivity and dependence on glycosaminoglycans, including heparan sulfate (HS) [12]. This dependence on glycosaminoglycans for initiating infection does not recapitulate wild-type RSV infection, as HS is absent in human airway epithelial cells and infection occurs mainly via specific receptor binding [43]. These G protein modifications affect RSV infection, permissibility, and the host response to infection. Thus, it is important to consider the cell type used for RSV propagation, as continuous cells are not necessarily interchangeable with respect to their permissiveness, their sensitivity to different RSV strains, or the host cell response to infection.

Differences in RSV susceptibility and replication exist between continuous cell lines and normal human cells, particularly between infection with laboratory isolates compared to clinical RSV isolates. A useful immortalized but non-tumorigenic human respiratory epithelial cell line is the BEAS-2B cell line, which has been used to compare RSV infection and host cell responses [44]. BEAS-2B cells are highly permissive to RSV infection but have restricted RSV replication, which has been linked to their antiviral response that is associated with type I and III IFN expression [45]. RSV-infected BEAS-2B cells respond similarly to RSV-infected primary human respiratory epithelial cells in which the differential expression of type I and III IFNs, ISGs, and proinflammatory cytokines is expressed [46]. The most susceptible to RSV infection are Vero cells, which lack the expression of type I IFNs while retaining type I receptors [47]. Differences in RSV susceptibility are not exclusive to cell lines. Genetic differences in mice have also revealed strain-dependent differences. For example, RSV titers are considerably higher in AKR/J mice, which are permissive compared with C57BL/6J mice, which are resistant [48]. Some inbred mice, e.g., BALB/c mice, have silenced Mx-1 genes, making them more susceptible to viral infections [49]. The interferon-inducible Mx protein is responsible for a specific antiviral state against virus infection [50]. Thus, it is important to consider genetic differences and how this may affect the results.

## 3. RSV Fitness Is Strain Dependent

The findings that RSV is altered by the cell line or cell type during infection show that the host cell can modify viral fitness and permissibility that can be dependent on the infecting strain. The strain of RSV is also important to consider. For example, RSV strain A2 was first isolated in 1961 from the lower respiratory tract of an infant [51] and is the predominant strain used for the development of RSV vaccine candidates. There have been limited in vitro or immunology studies examined for RSV strain B (RSV/B) despite a similar circulation to strain A [51,52,53,54]. RSV strain differences are a determinant of disease phenotypes and severity [55]; thus, it is important to study strain differences in response to infection. RSV line 19 is an RSV A strain that was first isolated from an infant with respiratory illness [56]. RSV A strains (A2, Long) have been widely used laboratory strains used in small animal studies of RSV pathogenesis but inadequately emulate RSV disease. In contrast, the RSV clinical strain, i.e., Line19, induces airway mucus expression in BALB/c mice, as well as lung dysfunction and mucin expression [57], and the chimeric Line19F has improved thermostability and further induces a mucogenic response in mice [58]. Another RSV clinical strain commonly used for clinical trials is the Memphis-37 strain, which is an A strain isolated from a child with bronchiolitis [59]. Memphis-37 has been used to study RSV pathogenesis and test vaccines and RSV inhibitors in human challenge studies [51,60,61]. Although uncommonly used in pre-clinical studies or clinical trials, the two circulating RSV strains ON (RSV A) and BA (RSV B) contain duplications in the G protein, which likely improve viral fitness [60,62]. It is evident that an over-reliance on one RSV strain, which inadequately recapitulates RSV disease in mice (e.g., A2), may overstate or understate drug or vaccine efficacy.

## 4. G protein Functions as the Attachment and Immune Modifier Protein

Many RSV proteins can modify the host cell response to infection [37]. RSV interacts with the host to create a favorable environment for virus replication and transmission ultimately making the host more susceptible to infection. For example, the G protein has attributes that contribute to mimicry and immune evasion [63,64,65,66,67]. The soluble form of the G protein differs from the membrane-bound form in its oligomeric state but remains capable of binding to cell surface glycosaminoglycans [68]. An excess of the G protein is expressed as a soluble G in a form that functions as an antigenic decoy, and this molecule retains the same characteristics as Gm based on glycosylation and antibody reactivity [69]. The membrane-bound G protein is a type II glycosylated membrane protein that has an extracellular ectodomain containing a central conserved domain (CCD) with four cysteine residues that are highly conserved in all RSV isolates. The CCD contains a CX3C chemokine motif (amino acids 182–186) that facilitates RSV attachment to susceptible cells bearing a CX3C chemokine receptor, CX3CR1 [22]. CX3CR1 mimicry by the G protein facilitates RSV infection and may alter CX3CL1 (fractalkine) chemotaxis of human and mouse leukocytes [22,70]. Expression of the G protein during RSV infection of mice has also been shown to decrease the number of activated and RSV-specific pulmonary CX3CR1+ T cells, as well as natural killer (NK) cells [70,71]. These studies suggest that the RSV G protein can modulate immune responses. Additionally, there is a link between the RSV G protein, other RSV proteins, and IFN expression, particularly for NS proteins [72,73,74]. As type I IFNs typically sensitize infected cells to programmed cell death, type III IFNs induce an anti-viral state. RSV infection induces high expression levels of IFNλ, which in one recently study was shown as protective [75], while another associated type III IFNs with more severe disease in children [76]. Thus, the RSV G protein may affect virus replication, transmission, immune responses, and disease.

## 5. Conclusions

Despite the several decades since RSV was first identified, there remains no safe and approved vaccine [77]. Prophylaxis is restricted to high-risk infants and has modest efficacy, and there is a lack of post-exposure therapeutic options. The chief reason for this lag is our incomplete understanding of RSV infection and disease. Current advances have led to the development of several promising vaccines and drug candidates. However, it is likely that more than one type of RSV vaccine will be needed to immunize those who could benefit from vaccination. In addition, more translational RSV studies need to be performed using primary or normal human cell lines and types infected with clinically relevant RSV strains to better understand the features contributing to replication, infectivity, transmission, and disease pathogenesis. It will also be important to consider vaccines and treatments that target both the RSV F and G proteins, as several preclinical studies have shown that anti-G protein antibodies both neutralize RSV infection in vivo and modify RSV disease [63,78,79,80,81,82,83,84], as well as human airway epithelial cells.
